# Supplementary material for: Multi‐dataset identification of innovative feature genes and molecular mechanisms in keratoconus
Source: J Cell Mol Med. 2024 Sep 19;28(18):e70079. doi: 10.1111/jcmm.70079 (PMC11412914; doi:10.1111/jcmm.70079)
Supplement: Supplementary file 2 — Material S2. [file JCMM-28-e70079-s002.zip › 7.GSEA analysis/3.DBNDD1/KEGG_TIGHT_JUNCTION.html]

Details for gene set KEGG\_TIGHT\_JUNCTION[GSEA]

|  || Dataset | DBNDD1.group.cls#h\_versus\_l |
| Phenotype | group.cls#h\_versus\_l |
| Upregulated in class | l |
| GeneSet | KEGG\_TIGHT\_JUNCTION |
| Enrichment Score (ES) | -0.34835625 |
| Normalized Enrichment Score (NES) | -1.2895343 |
| Nominal p-value | 0.1290944 |
| FDR q-value | 0.7609475 |
| FWER p-Value | 0.965 |
Table: GSEA Results Summary

  

Fig 1: Enrichment plot: KEGG\_TIGHT\_JUNCTION      
 Profile of the Running ES Score & Positions of GeneSet Members on the Rank Ordered List

  

| SYMBOL | TITLE | RANK IN GENE LIST | RANK METRIC SCORE | RUNNING ES | CORE ENRICHMENT || 1 | PPP2R2B | na | 705 | 0.414 | 0.0090 | No |
| 2 | CLDN5 | na | 1062 | 0.375 | 0.0253 | No |
| 3 | MYH13 | na | 1911 | 0.306 | 0.0225 | No |
| 4 | CLDN22 | na | 2892 | 0.256 | 0.0123 | No |
| 5 | MYH15 | na | 2956 | 0.253 | 0.0284 | No |
| 6 | RAB3B | na | 3189 | 0.244 | 0.0390 | No |
| 7 | MPDZ | na | 4273 | 0.204 | 0.0222 | No |
| 8 | CLDN23 | na | 4736 | 0.189 | 0.0222 | No |
| 9 | MYL5 | na | 4993 | 0.181 | 0.0276 | No |
| 10 | CLDN9 | na | 5115 | 0.176 | 0.0366 | No |
| 11 | PRKCG | na | 6020 | 0.150 | 0.0211 | No |
| 12 | MYH4 | na | 6442 | 0.138 | 0.0187 | No |
| 13 | PRKCQ | na | 6488 | 0.137 | 0.0271 | No |
| 14 | CLDN19 | na | 7495 | 0.114 | 0.0061 | No |
| 15 | MYH11 | na | 7776 | 0.108 | 0.0057 | No |
| 16 | CLDN20 | na | 7861 | 0.106 | 0.0108 | No |
| 17 | PPP2R1B | na | 8784 | 0.089 | -0.0096 | No |
| 18 | PARD6G | na | 8803 | 0.089 | -0.0038 | No |
| 19 | CLDN7 | na | 9113 | 0.084 | -0.0068 | No |
| 20 | AKT2 | na | 9388 | 0.080 | -0.0090 | No |
| 21 | AMOTL1 | na | 9811 | 0.074 | -0.0160 | No |
| 22 | OCLN | na | 9867 | 0.073 | -0.0124 | No |
| 23 | CLDN11 | na | 9992 | 0.071 | -0.0109 | No |
| 24 | ASH1L | na | 10289 | 0.067 | -0.0147 | No |
| 25 | EXOC3 | na | 10304 | 0.067 | -0.0103 | No |
| 26 | SYMPK | na | 10626 | 0.063 | -0.0152 | No |
| 27 | PTEN | na | 10959 | 0.059 | -0.0206 | No |
| 28 | MYH14 | na | 11418 | 0.053 | -0.0301 | No |
| 29 | TJP1 | na | 11569 | 0.052 | -0.0308 | No |
| 30 | CTNNA2 | na | 11866 | 0.048 | -0.0359 | No |
| 31 | EPB41L1 | na | 12099 | 0.046 | -0.0393 | No |
| 32 | SPTAN1 | na | 12139 | 0.046 | -0.0372 | No |
| 33 | PARD6A | na | 12250 | 0.045 | -0.0372 | No |
| 34 | PRKCH | na | 12623 | 0.042 | -0.0450 | No |
| 35 | ACTN4 | na | 12890 | 0.039 | -0.0500 | No |
| 36 | CLDN10 | na | 13201 | 0.036 | -0.0563 | No |
| 37 | CTTN | na | 13720 | 0.032 | -0.0690 | No |
| 38 | KRAS | na | 14127 | 0.029 | -0.0787 | No |
| 39 | PRKCZ | na | 14131 | 0.029 | -0.0767 | No |
| 40 | GNAI3 | na | 14246 | 0.028 | -0.0780 | No |
| 41 | ACTB | na | 14636 | 0.025 | -0.0875 | No |
| 42 | MYL9 | na | 14830 | 0.024 | -0.0913 | No |
| 43 | LLGL1 | na | 14905 | 0.024 | -0.0918 | No |
| 44 | LLGL2 | na | 14955 | 0.023 | -0.0916 | No |
| 45 | CLDN16 | na | 15149 | 0.022 | -0.0956 | No |
| 46 | GNAI2 | na | 15169 | 0.022 | -0.0946 | No |
| 47 | CLDN8 | na | 15262 | 0.021 | -0.0958 | No |
| 48 | MYH2 | na | 15817 | 0.017 | -0.1106 | No |
| 49 | PPP2R2C | na | 15941 | 0.016 | -0.1130 | No |
| 50 | EXOC4 | na | 16134 | 0.015 | -0.1175 | No |
| 51 | PARD3 | na | 16208 | 0.014 | -0.1186 | No |
| 52 | CSNK2A1 | na | 16316 | 0.014 | -0.1207 | No |
| 53 | CTNNB1 | na | 16378 | 0.013 | -0.1215 | No |
| 54 | CLDN3 | na | 16669 | 0.011 | -0.1291 | No |
| 55 | CRB3 | na | 16831 | 0.011 | -0.1330 | No |
| 56 | PPP2R2D | na | 16913 | 0.010 | -0.1347 | No |
| 57 | TJP2 | na | 16934 | 0.010 | -0.1345 | No |
| 58 | MYL12B | na | 17399 | 0.007 | -0.1475 | No |
| 59 | ACTN2 | na | 17576 | 0.006 | -0.1521 | No |
| 60 | MYL12A | na | 17584 | 0.006 | -0.1519 | No |
| 61 | CSNK2A2 | na | 17761 | 0.005 | -0.1567 | No |
| 62 | RAB13 | na | 17922 | 0.003 | -0.1611 | No |
| 63 | PRKCI | na | 18135 | 0.002 | -0.1671 | No |
| 64 | ACTG1 | na | 18138 | 0.002 | -0.1670 | No |
| 65 | YBX3 | na | 18160 | 0.002 | -0.1675 | No |
| 66 | CTNNA1 | na | 18276 | 0.001 | -0.1708 | No |
| 67 | HRAS | na | 18984 | -0.004 | -0.1909 | No |
| 68 | AKT1 | na | 19066 | -0.005 | -0.1929 | No |
| 69 | RHOA | na | 19096 | -0.005 | -0.1934 | No |
| 70 | TJP3 | na | 19453 | -0.008 | -0.2031 | No |
| 71 | CSNK2B | na | 19607 | -0.010 | -0.2068 | No |
| 72 | PPP2R1A | na | 19616 | -0.010 | -0.2064 | No |
| 73 | CGN | na | 19693 | -0.010 | -0.2078 | No |
| 74 | EPB41 | na | 20062 | -0.014 | -0.2175 | No |
| 75 | MYH9 | na | 20342 | -0.016 | -0.2244 | No |
| 76 | PRKCD | na | 20754 | -0.020 | -0.2349 | No |
| 77 | MAGI3 | na | 20946 | -0.022 | -0.2389 | No |
| 78 | F11R | na | 20999 | -0.022 | -0.2388 | No |
| 79 | CDK4 | na | 21131 | -0.023 | -0.2410 | No |
| 80 | CLDN4 | na | 21176 | -0.024 | -0.2406 | No |
| 81 | PRKCA | na | 21412 | -0.026 | -0.2455 | No |
| 82 | YES1 | na | 21708 | -0.029 | -0.2520 | No |
| 83 | CDC42 | na | 22626 | -0.039 | -0.2758 | No |
| 84 | VAPA | na | 22714 | -0.040 | -0.2754 | No |
| 85 | RRAS | na | 23237 | -0.046 | -0.2873 | No |
| 86 | PPP2R2A | na | 23306 | -0.047 | -0.2859 | No |
| 87 | MYH3 | na | 23326 | -0.047 | -0.2831 | No |
| 88 | PPP2CA | na | 23696 | -0.052 | -0.2901 | No |
| 89 | MYH7 | na | 23841 | -0.054 | -0.2904 | No |
| 90 | PPP2CB | na | 24140 | -0.058 | -0.2949 | No |
| 91 | MYH10 | na | 24633 | -0.065 | -0.3045 | No |
| 92 | CLDN1 | na | 24654 | -0.066 | -0.3004 | No |
| 93 | RRAS2 | na | 24862 | -0.069 | -0.3015 | No |
| 94 | MYL2 | na | 25552 | -0.079 | -0.3158 | No |
| 95 | CASK | na | 25568 | -0.080 | -0.3106 | No |
| 96 | GNAI1 | na | 25682 | -0.082 | -0.3081 | No |
| 97 | NRAS | na | 27014 | -0.108 | -0.3389 | Yes |
| 98 | EPB41L3 | na | 27032 | -0.108 | -0.3317 | Yes |
| 99 | CTNNA3 | na | 27076 | -0.110 | -0.3252 | Yes |
| 100 | MAGI1 | na | 27668 | -0.123 | -0.3336 | Yes |
| 101 | MYL7 | na | 28100 | -0.133 | -0.3366 | Yes |
| 102 | TJAP1 | na | 28509 | -0.144 | -0.3382 | Yes |
| 103 | EPB41L2 | na | 28513 | -0.144 | -0.3281 | Yes |
| 104 | SRC | na | 28777 | -0.151 | -0.3250 | Yes |
| 105 | CLDN18 | na | 28929 | -0.155 | -0.3184 | Yes |
| 106 | MYH1 | na | 29359 | -0.168 | -0.3189 | Yes |
| 107 | MYH6 | na | 29406 | -0.169 | -0.3083 | Yes |
| 108 | MYH7B | na | 29462 | -0.171 | -0.2977 | Yes |
| 109 | CLDN6 | na | 30134 | -0.192 | -0.3035 | Yes |
| 110 | ACTN1 | na | 30298 | -0.197 | -0.2942 | Yes |
| 111 | CLDN15 | na | 30342 | -0.199 | -0.2814 | Yes |
| 112 | MAGI2 | na | 30803 | -0.214 | -0.2795 | Yes |
| 113 | PRKCE | na | 32256 | -0.280 | -0.3016 | Yes |
| 114 | PARD6B | na | 32493 | -0.293 | -0.2877 | Yes |
| 115 | CLDN17 | na | 32713 | -0.307 | -0.2723 | Yes |
| 116 | JAM2 | na | 32852 | -0.317 | -0.2538 | Yes |
| 117 | HCLS1 | na | 32856 | -0.317 | -0.2314 | Yes |
| 118 | MYL10 | na | 32938 | -0.324 | -0.2108 | Yes |
| 119 | CLDN14 | na | 33082 | -0.334 | -0.1912 | Yes |
| 120 | MYH8 | na | 33346 | -0.357 | -0.1735 | Yes |
| 121 | IGSF5 | na | 33408 | -0.362 | -0.1497 | Yes |
| 122 | AKT3 | na | 33656 | -0.385 | -0.1296 | Yes |
| 123 | MRAS | na | 33756 | -0.398 | -0.1042 | Yes |
| 124 | CLDN2 | na | 33855 | -0.411 | -0.0779 | Yes |
| 125 | ACTN3 | na | 33884 | -0.415 | -0.0493 | Yes |
| 126 | PRKCB | na | 34153 | -0.452 | -0.0251 | Yes |
| 127 | JAM3 | na | 34561 | -0.588 | 0.0048 | Yes |
Table: GSEA details [plain text format]

  

Fig 2: KEGG\_TIGHT\_JUNCTION      
 Blue-Pink O' Gram in the Space of the Analyzed GeneSet

  

Fig 3: KEGG\_TIGHT\_JUNCTION: Random ES distribution      
 Gene set null distribution of ES for **KEGG\_TIGHT\_JUNCTION**
